# Toolkit for integrating millimeter-sized microfluidic biomedical devices with multiple membranes and electrodes

**DOI:** 10.1038/s41378-025-00871-0

**Published:** 2025-02-27

**Authors:** Xudong Tao, Tobias E. Naegele, Etienne Rognin, Niamh Willis-Fox, Poppy Oldroyd, Chaoqun Dong, Stefany Kissovsky, Antonio Dominguez-Alfaro, Santiago Velasco-Bosom, Ronan Daly, George G. Malliaras

**Affiliations:** 1 Electrical Engineering Division, Department of Engineering, University of Cambridge, Cambridge, CB3 0FA UK; 2 Institute for Manufacturing, Department of Engineering, University of Cambridge, Cambridge, CB3 0FS UK

**Keywords:** Electrical and electronic engineering, Physics

## Abstract

In recent years, microfluidic systems have evolved to incorporate increasingly complex multi-layer and multi-material structures. While conventional 2-dimensional microfluidic systems are typically fabricated with lithographic techniques, the increase in system complexity necessitates a more versatile set of fabrication techniques. Similarly, although 3D printing can easily produce intricate microfluidic geometries, integrating multiple membranes and electrode components remains challenging. This study proposes a toolkit for fabricating free-standing 3-dimensional microfluidic systems for biomedical devices, incorporating flow channels, electrodes, and membranes. The fabrication techniques include molding separation using 3D printed molds, laser-based processing, and component assembly, each achieving micron resolution. Here, we introduce a novel approach to integrate membranes into microfluidics by directly curing elastomer-based microfluidics with the membrane through replica molding, while preserving membrane functionality by effectively removing elastomer residues through reactive ion etching. The resulting membrane-elastomer microfluidic component significantly simplifies the assembly of intricate microfluidic systems, reducing the device size to millimeter dimensions, suitable for implantable applications. The toolkit’s versatility is demonstrated by a redox flow iontophoretic drug delivery prototype at the millimeter scale, featuring two electrodes, four membranes, and four microfluidic channels.

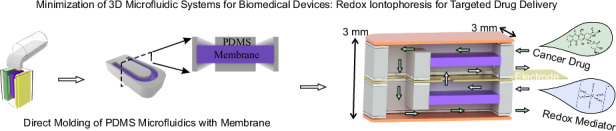

## Introduction

In recent years, microfluidic systems have emerged as a crucial technology with diverse applications across various fields including biomedical devices^1^. A recent trend in the field involves the integration of components such as electrodes^2^ and separation membranes^3^. This significantly extends their functionality in in vitro systems for drug discovery/toxicology^4,5^, electroporation platforms for cell transfection^6,7^, and iontophoresis devices for drug delivery^8,9^. However, fabrication of these microfluidic-based systems presents unique challenges, particularly when considering devices that are free-standing, small, and have intricate 3-dimensional structures^10^. Developing a standardized toolkit for fabricating such microfluidic systems would be beneficial to the field and enable portable and implantable formats.

Microfluidic systems can generally be fabricated using microfabrication, 3D printing, and replica molding^11,12^. Traditional microfabrication methods, such as lithography or nanoimprint, are effective for creating large sheets of two-dimensional microfluidic systems but face challenges in fabricating complex systems with integrated electrodes and separation membranes^13–15^. 3D printing and replica molding are simple, scalable, capable of high resolution, and compatible with polydimethylsiloxane (PDMS), a widely used biocompatible elastomer for microfluidics^12^. Although 3D-printed microfluidics have been extensively studied in biomedical applications, most reported devices are centimeter-sized and intended for in vitro use^16–20^. Miniaturizing microfluidic systems to millimeter size or smaller is essential for implantable applications to reduce the pain and distress to the animal^21^. While 3D printing techniques can achieve millimeter-scale devices, integrating membranes into such small systems remains a significant challenge. Recent advances in 3D-printed porous membranes in microfluidics have made substantial contributions to this field, though the authors noted limitations regarding the limited resin materials for the membranes and the necessity for human intervention (e.g., washing, wiping, and wicking) in multi-resin 3D printing processes^22^. Ion exchange membranes (IEM) are gaining attention in biomedical applications^23^. Unlike porous membrane materials, 3D printing IEM membrane is challenging because these materials must be properly hydrated to avoid issues with drying or flooding^24–26^. Direct incorporation of commercial membranes^13^ remains the most effective method for integrating IEM into microfluidics. Techniques such as heating^27,28^ and plasma-assisted bonding^29^ are unsuitable for IEM, as they cause IEM deformation e.g., warping and distortion. Bonding methods that use liquid glue^30,31^ also pose challenges in localizing the glued area and controlling the amount applied, particularly when device dimensions are reduced to the millimeter scale, potentially resulting in blocked membrane regions. Therefore, developing a toolkit for integrating membrane into millimeter-sized microfluidic systems is essential.

This study proposes a novel approach to directly integrate membrane with elastomer-based microfluidics using 3D-printed molds. Focusing on the minimization of intricate microfluidic systems suitable for implantable applications such as redox flow iontophoresis^32^, we extend the capability of replica molding by combining it with laser cutting and component assembly. This method enables the facile fabrication of compact devices (3 mm × 3 mm × 10 mm) that seamlessly integrate four microfluidics with two electrodes and four separation membranes. We demonstrate this approach through the design, fabrication, and validation of a prototype drug delivery device based on redox flow iontophoresis.

## Toolkit for microfluidic-based bioelectronics

**Fig. 1:**
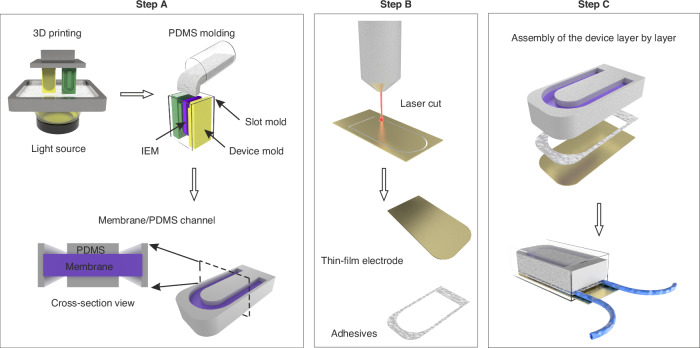
The toolkit for microfluidic iontophoresis (see Fig. S1 for details, Supplementary Information). Step A: The membrane is slid into the device mold, followed by filling and curing with PDMS to integrate the microfluidic channels and membrane; Step B: Both thin-film flexible electrodes and bulk electrodes can be machined using a laser cutter, along with the adhesives; Step C: A semiconductor-level aligner is employed for layer-by-layer integration (top: alignment of three components; bottom: integrated device with tube connection)

The essential toolkit for microfluidic-based bioelectronics capable of integrating electrodes and separation membranes is shown in Fig. 1. The processing techniques include 3D printing for mold fabrication, replica molding of PDMS with separation membranes (step A), laser cutting of various components (step B), and device assembly using an aligner bonder (step C). These processes constitute a versatile toolkit that can be processed batch-to-batch to mass-produce millimeter-size microfluidic systems with diverse complex geometries integrating multiple separation membranes and electrodes.

Here we discuss the individual steps of the toolkit. The first step in the process is high-resolution stereolithography 3D printing to create microfluidic molds (Fig. 1 A). Both in-plane U-shape and cross-plane U-shape molds can be designed to fabricate multi-channel microfluidics incorporating multiple membranes and electrodes. Subsequently, the membrane sheet is inserted into the microfluidic mold, where PDMS is degassed for approximately 30 minutes with a rotary pump and cured at 55 °C for 2.5 h. To ensure easy demolding, a 2-µm thick parylene coating is deposited on the molds using chemical vapor deposition, which provides atomic-level conformal coating, uniformly covering all surfaces of the fine features of the molds. The anti-adhesive properties of parylene, attributed to the surface energy mismatch between PDMS and parylene, ensure smooth and trouble-free PDMS demolding^33^. The parylene-coated mold remains reusable and functional after 20 tests. Some polymer membranes, such as IEM, are prone to deform after repeated multiple cycles of drying and hydration^24–26^ (see Fig. S2a, Supplementary Information), complicating their integration into PDMS chips without leakage. Shaping the IEM can be accomplished by sandwiching a wet-state IEM between two 175-µm thick polyethylene terephthalate (PET) sheets, followed by laser cutting. However, integrating the dried, and thus deformed, IEM on microfluidics poses challenges. This study addressed this issue by directly integrating the IEM membrane with uncured PDMS using 3D-printed microfluidics molds. The cured PDMS-based microfluidics are robust enough to prevent membrane deformation, although the effectiveness depends on the elastomer channel thickness (see Fig. S3, Supplementary Information). This approach greatly simplifies the integration process.

**Fig. 2:**
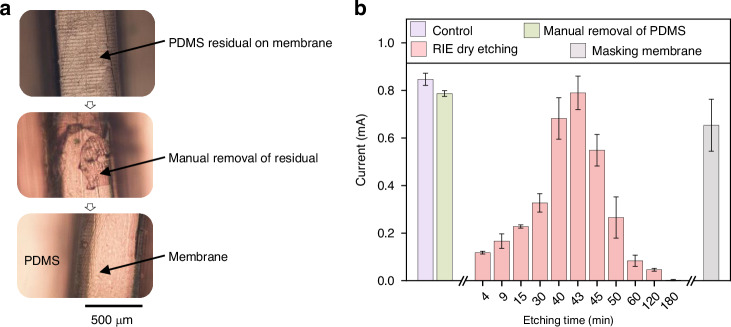
Characterization of the membrane. **a** Top: membrane with PDMS residuals after molding, middle: manual removal of PDMS, bottom: completely freed membrane; **b** Characterization of membrane permeability using the described sodium ferrocyanide redox approach. As shown in Fig. S2d (Supplementary Information), the microfluidic system and the counter electrode Ag/AgCl are immersed in NaCl solution, while the microfluidic channel is filled with sodium ferrocyanide redox solution. As voltage is applied, higher currents indicate better membrane permeability. Optical images of the IEM at various RIE times are shown in Fig. S2c. The control devices were assembled using a different fabrication approach which does not result in membrane blockage (see Methods). The error bars denote the standard deviation measured with three identical devices

However, curing PDMS in the presence of a membrane can result in a thin layer of PDMS on the membrane that may potentially obstruct the membrane during device usage. Ensuring precise control over the uniformity of this PDMS residual layer is crucial during the molding process, as the effective removal of the PDMS residual depends on it. Moreover, any non-uniformity of the residual layer could cause damage to the membrane during the removal process. Therefore, the device molds are inserted into a “slot” mold before filling with PDMS to accurately control the thickness of the residual PDMS layer (note: clamping microfluidic molds with clips cannot achieve the same level of precision control over the PDMS residuals). The thin PDMS layer on the membrane can be manually removed using a tweezer (Fig. 2a). However, manually pulling or peeling off the residual PDMS layer with tweezers risks damaging the membrane and requires excessive human intervention, making this approach impractical for scale-up production. Two alternative solutions have been identified: (1) Before molding, masking the membrane with water-soluble polymer films such as PVA-based 3 M 5414 tape, which can be removed with water after PDMS curing; (2) Removing the PDMS residuals using reactive ion etching (RIE)^34,35^. The masking approach still leaves a residual PDMS layer that requires tweezer removal. While the inclusion of a water-soluble film as an interlayer makes peeling the layer off easier, it still involves human intervention, limiting the effectiveness and scalability of this method. More importantly, the tweezer-based method becomes impractical when the device or membrane size is reduced below the width of the tweezer tip. The RIE method is recommended, as it eliminates the need for tweezers and allows for the simultaneous processing of multiple devices in the RIE chamber. This makes it a more scalable solution for producing compact microfluidic devices.

The membrane permeability is characterized using a redox-based electrode configuration, as reported in ref. ^32^. Applying a voltage causes the redox reaction of the molecule, with the electric current being restricted by the membrane’s permeability. A low electric current therefore indicates that the membrane is blocked by the PDMS residuals. In Fig. 2b, it is confirmed that all three solutions (i.e., manual removal, masking, and RIE) effectively remove the PDMS residuals, with the RIE method being preferred for greater simplicity and higher throughput. It should be noted that there exists an optimal etching time in RIE: insufficient etching time results in PDMS residue remaining; while exceeding the optimal time leads to membrane damage, compromising its functionality. The optimized etching time of 43 minutes indicates a residual PDMS layer thickness of 14.7 μm on the membrane. This etching time has been consistently validated across 18 samples from 4 different batches, demonstrating the reproducibility of this method.

The electrode and adhesive sheets can be shaped using a laser cutter or a cutting plotter (Fig. 1b). The cutting plotter enables the shaping of heat-sensitive materials while the laser cutter offers higher resolution down to the micron-meter level. Afterward, the produced membrane-PDMS microfluidics and electrodes can be arbitrarily stacked layer-by-layer (using dry-state double-sided water-resistant adhesive films) to create 3-dimensional microfluidic systems. Using dry adhesive films instead of liquid adhesives offers advantages such as preventing bleeding of adhesive into the membrane and microfluidic channel area, while also increasing the overall ratio of membrane and channel to bonding site. Hence, this overcomes limitations on channel size, particularly in the case of microfluidics with multiple membranes. Semiconductor-level aligner, commonly employed for printed circuit board assembly, can be utilized for bonding/stacking, providing micron-meter positioning accuracy and controlled application of bonding pressure. The aligner enables the simultaneous assembly of multiple devices with each step in a batch-to-batch fashion. Finally, tubing and electrical wiring can be attached to the assembled device to facilitate fluidic and electrical interfacing. Partially encapsulating the final device in a biocompatible UV curing cyanoacrylate layer can improve mechanical stability, at the expense of some flexibility of the device.

To assess the pressure tolerance of the device assembled using our toolkit, we constructed a test structure featuring an in-plane U shape channel, assembled layer-by-layer with membrane-PDMS microfluidics, adhesives, electrode substrate, and a subsequent cyanoacrylate layer (refer to Fig. S2e in Supplementary Information). The burst pressure of this test structure was determined using a setup incorporating a syringe pump to increasing the liquid pressure within the channel until leakage was detected. Our observation revealed a burst pressure of (372 ± 19) mBar, with the interface between the PDMS channels and the membrane failing under this pressure. This pressure level aligns well with the typical range observed in implantable microfluidics^10^.

## Microfluidic iontophoresis device

**Fig. 3:**
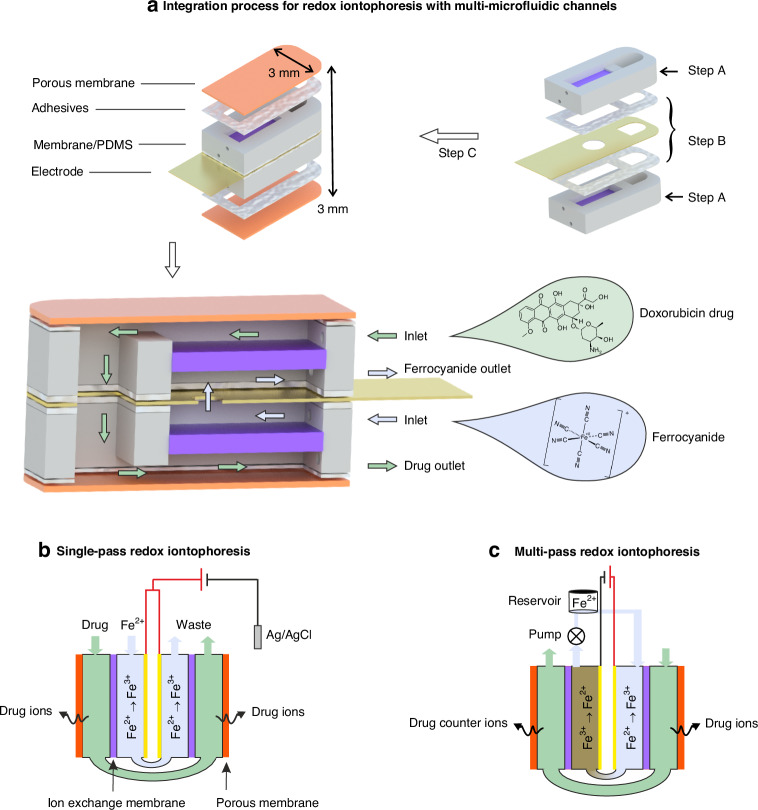
Microfluidic iontophoresis: structure and mechanism. **a** Fabrication processes of four-channel microfluidic iontophoresis with cross-section U-shape geometry in a dimension of 3 mm × 3 mm × 10 mm (the membrane/PDMS microfluidics are fabricated using step A, while the thin-film electrode/adhesive/porous membrane are fabricated using step B, see Figs. 1 and S1 for details); **b**, **c** Mechanism of the device for drug delivery: cross-sectional view

Implantable redox flow iontophoresis necessitates a compact design, featuring both a redox flow channel and a drug channel, making it an exemplary application for showcasing the versatility of our toolkit in handling complex and compact geometries. Redox flow iontophoresis^32^ incorporates a redox flow channel to physically separate the metal electrode from the drug solution. This configuration enables the continuous extraction of drug counter ions across the membrane from the drug channel to the redox channel, consequently releasing drug molecules outside the device. In our previous study^32^, this concept was validated using a 3D printed geometry featuring two in-plan U-shaped microfluidics with one-side drug delivery. This toolkit can effectively fabricate this complex device with several advantages: (1) further reduction in the device size; (2) two-side drug delivery. Instead of the in-plane U-shape design, a cross-section U-shape microfluidic system can be realized using our toolkit, as illustrated in Fig. 3a. Thin metallic films were sputtered on both sides of the flexible PET sheet as electrodes, and then shaped with two holes: one for the internal fluid and another for the external fluid. The membrane-PDMS microfluidics was molded with an open window at the tip (see Fig. 3a and Fig. S1a in Supplementary Information). After stacking two membrane-PDMS microfluidic layers on both sides of the flexible electrode, a commercial 10 μm thick cellulose-based porous dialysis membrane sheet (Spectra/Por®, see Fig. S2b, Supplementary Information) was affixed on top of the PDMS channels using dry-state adhesives, followed by the connection of tubes and cables. The device now incorporates four channels, two electrodes, and four membranes. The inner two IEM (shown in purple in Fig. 3a) separate the redox species from the drug solution, while the outer two porous membranes (shown in orange in Fig. 3a) facilitate the external release of drug molecules. Utilizing our toolkit, the size of this compact system can be significantly reduced to a dimension of 3 mm × 3 mm × 10 mm, rendering it suitable for implantable applications, e.g., a 2–10 mm diameter cranial window for rodent experiments^36,37^.

The fabricated device can be utilized under two configurations, as depicted in Fig. 3b, c. In the single-pass case, the device is positively charged. As a continuous supply of fresh ferrocyanide redox solution flows through the redox channel, it undergoes oxidation from Fe^2+^ to Fe^3+^, maintaining a positively charged state within the redox channel. This facilitates the extraction of negatively charged drug counter ions from the adjacent drug channels via the IEM. As a result, positively charged drug ions are released through the porous membrane to the outside of the device. To facilitate this process, an Ag/AgCl electrode^38^ serves as a counter electrode to supply Cl^−^ ions to neutralize the positively charged drug ions released. In terms of the multi-pass case, the flexible electrode is polarized positively on one side and negatively on the other. The internal channel forms a closed-loop fluid flow to recycle the redox solution within the redox channel, facilitating the electrochemical reaction: the oxidation reaction occurs on the positively charged side; conversely, the reduction reaction occurs on the negatively charged side. Consequently, the drug ions are released on one side while the drug counter ions are released on the other side. By switching the polarized sides positively and negatively, drug ions can be released alternatively on both sides.

**Fig. 4:**
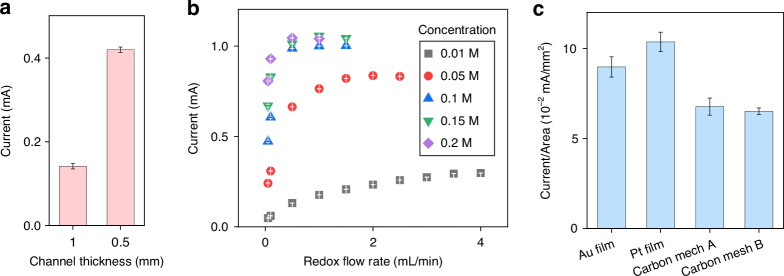
Impact of flow channel thickness, redox species, and electrode materials on device performance. **a** Effect of channel thickness on current response under 1 V; **b** Optimizing the flow rate and concentration of the redox solution on the current response under 1 V (the maximum flow rate and pressure limit for the redox channel of microfluidics are 5 mL/min and 372 mBar, respectively); **c** Current response of redox iontophoresis with different electrode materials under 1 V

The efficiency of electron transfer is crucial for the performance of redox-based devices^39^, as electron transfer in redox reactions corresponds to the drug delivery efficiency in redox flow iontophoresis. The redox reaction occurs at the electrode-electrolyte interface, which is influenced by the rate of reactant molecule diffusion towards the electrode surface. This diffusion rate depends on various factors, such as the strength of the electric field^40^, beyond which the electric field becomes negligible. Reducing the channel thickness may be an effective way to enhance the efficiency of electron transfer and thus the device performance. As expected, reducing the channel thickness from 1 mm to 0.5 mm results in an increase in current from 0.14 mA to 0.42 mA (Fig. 4a), with an 85% decrease in impedance and a 32% increase in the delivery rate (refer to Fig. S4, Supplementary Information). The flow rate and concentration of the redox solution are also critical factors affecting electron transfer and the overall delivery efficiency of the device. As illustrated in Fig. 4b, a higher flow rate is required to achieve equilibrium at low redox electrolyte concentrations, due to the insufficient availability of redox species for the reaction. Each concentration eventually reaches a specific equilibrium, beyond which the current ceases to increase. Once the redox concentration exceeds 0.1 M, the current approaches equilibrium.

**Fig. 5:**
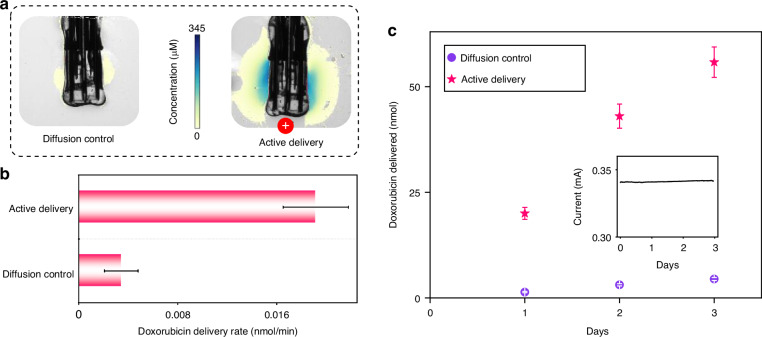
Device characterization. **a** Imaging of methylene blue delivery into an agarose brain phantom (the plume demonstrates how the delivered molecules diffuse, in the single-pass redox case); **b** The drug delivery rate in the single-pass redox case; **c** Long-term drug delivery in the multi-pass redox case

Selecting appropriate electrode materials is crucial for the performance of redox flow iontophoresis, concerning delivery efficiency and device stability. Our toolkit supports the use of both bulk sheet and thin film electrodes, such as carbon meshes, gold (Au) and platinum (Pt) films. Porous carbon-based mesh materials are popular in redox flow batteries due to their low cost^41^, high surface area (refer to Fig. S5a, Supplementary Information), and efficient electron transfer^42^, suggesting their potential use in iontophoretic devices. The integration of carbon mesh on flexible substrates requires a conductive tape (e.g., the C tape used in microfluidics^43^), resulting in high impedance (refer to Fig. S5b, Supplementary Information) due to the presence of adhesives on the C tape. Hence it reduces the current response and thus the efficiency of electron transfer in the redox reaction. In addition, the low wettability of bulk carbon mesh (i.e., carbon felt^44^) limits its reaction with electrolyte and the flow rate of the redox solution. Au and Pt are common electrode materials for the redox reaction of ferrocyanide/ferricyanide because of their stability^45^. Au (or Pt) coating on flexible polymer substrates can be fabricated through physical vapor deposition, and the fabricated flexible thin-film electrode can be integrated with PDMS-based microfluidics using an aligner. Although evaporated Au has greater sheet conductance (see Fig. S5b, Supplementary Information), its impedance is inferior because sputtered Pt possesses a much rougher surface morphology in our case, resulting in a greater surface area and thus a better current response (refer to Fig. S5b). This indicates greater efficiency of electron transfers due to a high electrode-electrolyte interface for the reaction^46^. In Fig. S6 (Supplementary Information), the Au electrode is observed to be consumed/delaminated after 8 h, although the Au element in the waste from the redox outlet is insufficient to be detected by ICP-OES. The Pt electrode does not exhibit this kind of consumption/delamination phenomenon after 8 h. The stability of the Pt electrode is further confirmed by 3-day testing in Fig. 5c. Hence, the Pt electrode is highly recommended for its high efficiency and long-term stability.

The delivery efficiency of the device can be assessed both visually and quantitatively. Visually, methylene blue molecules are used (Fig. 5a), while the quantitative assessment involves the delivery of the cancer drug doxorubicin (Fig. 5b). In the single-pass redox case (Fig. 3b), the device, along with an Ag/AgCl counter electrode positioned in parallel, was immersed in an agarose gel brain phantom^47^. A 0.2 M sodium ferrocyanide solution was flushed through the redox channel at a flow rate of 0.3 mL/min, while a 0.63 mM methylene blue solution was pumped through the drug channel at 1 mL/h. Upon applying a voltage of 0.5 V, anions from the drug channel were extracted into the redox channel, and cationic methylene blue molecules were delivered into the agarose gel, as visually confirmed in Fig. 5a (see Fig. S3 for the experimental setup). A clear contrast between the active device and the diffusion control demonstrates the functionality of the device, indicating that our toolkit can effectively be used to fabricate complex and functional microfluidic iontophoretic systems. For further quantification of delivery efficiency, the cancer drug doxorubicin was utilized. The redox and drug solutions were flushed through the device at flow rates of 0.3 mL/min and 1 mL/h, respectively, with the device and Ag/AgCl counter electrode submerged in a 100 mM NaCl solution (see Fig. S2d). Applying a voltage of 1 V activated the device, allowing cationic doxorubicin molecules to be delivered into the NaCl solution. The concentration of doxorubicin in the NaCl solution was quantified using fluorescence measurements with a Tecan Spark plate reader, employing an excitation wavelength of 480 nm and a readout at 590 nm. The results demonstrated a significantly enhanced drug delivery efficiency compared to the control group relying on passive diffusion (refer to Fig. 5b). A linear relationship is estimated between the quantity of delivered drug and the injected charge (refer to Fig. S7a, Supplementary Information). This suggests a correlation between the injected charge and drug delivery, consistent with previous studies^48,49^. Additionally, it is confirmed that there is negligible diffusion of redox species outside of the device (refer to Fig. S7b, c, UV-Vis and ICP-OES, Supplementary Information). While these data offer a preliminary estimate of drug delivery based on injected charges for future in vivo experiments, it is crucial to thoroughly characterize each individual device beforehand to ensure accurate delivery of the cancer drug in vivo.

In contrast to the single-pass case, the multi-pass redox flow iontophoresis does not require the replacement of the Ag/AgCl counter electrode, enabling long-term stability testing. Over several days, a significant increase in drug delivery was observed under a 0.34 mA current compared to the diffusion control (refer to Fig. 5c). However, bubbles were observed in the redox tube at approximately 2.5 days, which may be attributed to a pH drop in the redox solution (refer to Fig. S8, Supplementary Information). Refilling the redox solution every 2 days is recommended. The chemistry of the bubble is currently under investigation since the amount of the bubble is too small to detect. Assumptions regarding this phenomenon are detailed in the Supplementary section.

## Discussion

Our toolkit facilitates the production of compact devices featuring multiple microfluidic channels, membranes, and electrodes with diverse geometries. Compared to our previous study (device size: 4 mm diameter × 16 mm length with two channels^32^), this work reduces the device size to 3 mm × 3 mm × 10 mm (with four channels) while maintaining a similar delivery rate (see Fig. S9b, Supplementary Information). Notably, this work doubles the microfluidic channels, enabling two-sided drug delivery and multi-pass redox iontophoresis for long-term delivery. Device minimization necessitates reducing the channel thickness to the smallest feasible dimension. Using this toolkit, the minimum channel dimensions are constrained to ~0.3 mm in thickness and ~1.8 mm in width. The thickness is constrained to sustain the internal strain of the IEM membrane (AMX-fg, *EURODIA INDUSTRIE SAS*), which could affect the stacking process during assembly. It is noted that the PDMS channels on either side of the IEM do not need to be symmetric to stabilize the IEM and prevent deformation (e.g., 1 mm thick on one side and 0.2 mm thick on the other, see Fig. S3b, Supplementary Information). To simplify membrane insertion into the mold and avoid using a microscope during fabrication, the minimum channel width was set to 1.8 mm. Our fabrication approach can produce redox flow iontophoresis devices of various sizes, suitable for wearable and implantable applications. In addition to compact implants (3 mm × 3 mm × 10 mm, with cross-section U-shape geometry), our toolkit enables the fabrication of larger-area devices (1 cm × 1 cm × 3 mm, with in-plan wave-U-shape geometry, see Fig. S9, Supplementary Information). Featuring a substantial electrode area, this device exhibits a very high delivery efficiency (0.22 nmol min^−1^ for doxorubicin under 1 V). The large-area devices are suitable for wearable applications such as skin wound healing^50^, and chronic skin cancer treatment^51^.

In terms of the device configuration, the single-pass device requires a continuous flow of the redox and drug solutions through the device to ensure a controlled concentration and thus consistent drug delivery efficiency. However, this setup results in material wastage and necessitates frequent hospital visits for treatment. In contrast, multi-pass redox flow iontophoresis (Fig. 3c) has the potential to enhance the device’s usability for portable and wearable applications. Our prototype device makes the concept of Hospital at Home care feasible by integrating portable liquid pumps and a power system, similar to the SynchroMed™ pain pump (refer to Fig. S10, Supplementary Information). Both the redox and drug fluid flows operate in a closed cycle using an external pump system, ensuring the maintenance of the redox reaction in the redox channel and maximizing the utilization of the drug solution. Another significant advantage of the multi-pass redox flow iontophoresis is the elimination of the impact of skin resistance in real implantable applications. Skin resistance impacts the efficiency of drug delivery and can cause skin irritation^52,53^. Unlike the single-pass device necessitating an Ag/AgCl electrode (e.g., DENIS10026, Spes Medica) attached to the skin, the multi-pass redox flow iontophoresis confines the counter electrode within the implantable device. This arrangement avoids the drop of voltage caused by skin resistance and eliminates the risk of tissue irritation by two electrodes, as they are internal electrodes within the device. It should be noted that there is an internal electrical circuit through the redox channel. The internal circuit within the device provides a protective mechanism to prevent burning of the surrounding tissue, however, this also indicates that some injected charges do not effectively contribute to the drug delivery process (which should be through the external electrical circuit of the device), confirmed by a significantly higher current response but with a lower delivery rate (refer to Fig. S11, Supplementary Information). This is because the internal path, specifically, the ferrocyanide solution, has significantly lower impedance, making it more favorable than the external path, which consists of the IEM, drug solution, porous membrane, and NaCl solution. Further studies on electrode circuit design are being conducted to enhance the effectiveness of the injected charge for drug delivery. Furthermore, multi-pass redox flow iontophoresis can deliver both cationic and anionic drugs simultaneously (refer to Fig. S12, Supplementary Information), opening up opportunities for multi-type drug delivery. This potential capability could lead to synergistic therapeutic effects^54^ in areas such as cancer therapy, chronic treatment, and neurological disorders.

## Conclusions

This study proposed a versatile toolkit for fabricating compact, free-standing, 3-dimensional microfluidic systems featuring multiple membranes and electrodes for biomedical applications. The processing techniques encompass 3D printing for mold fabrication, replica molding of PDMS with membrane, laser cutting of electrodes and adhesives, and device assembly using an aligner. The membrane can be integrated directly with elastomer-based microfluidics using a molding technique, thereby significantly simplifying the device assembly process. This toolkit enables batch-to-batch processing to scale up the production of intricate structures, and operate at sub-millimeter resolution thus significantly compacting the device size to millimeter dimension, rendering it suitable for implantable applications.

The toolkit was successfully validated using a redox flow iontophoretic device featuring a cross-section U-shape microfluidics design, which achieved dimensions of 3 mm × 3 mm × 10 mm suitable for implantable applications. The fabricated device shows promise for multi-type drug delivery offering synergistic therapeutic effects, and can potentially serve as a wearable or implantable Hospital at Home solution in areas such as cancer therapy, chronic treatment, and neurological disorders.

## Methods

### Materials and processing

The 3D printer employed in this study was a high-resolution stereolithography system (MAX X, Asiga®). The IEM and the porous membranes were made from aromatic polystyrene/divinylbenzene-based materials (AMX-fg, EURODIA INDUSTRIE SAS) and cellulose-based dialysis membranes (3.5 kD, Spectra/Por®), respectively. The PET sheets were 175-µm thick polyethylene terephthalate (Hi-Fi Industrial Film Ltd.). Laser cutting was performed using a VLS CO_2_ Laser (Universal®) at 5% power, 2% speed, 1000 PPI, and a 1.3 mm *Z*-axis setting, with two passes. Parylene coatings were deposited via an SCS PDS 2010 lab coater, with a dichloro-p-cyclophane precursor (Galentis, Italy) under conditions of 690 °C furnace temperature, 135 mBar chamber pressure, and 175 °C vaporizer settings. The RIE (Oxford Instruments) was conducted at 250 W, with 40 sccm of SF_6_ and 10 sccm of O_2_, yielding a PDMS etching rate of 0.34 μm/min. Alignment was carried out using a Picoplacer machine (Finetech GmbH & Co. KG). Biocompatible UV-curing cyanoacrylate adhesives (4305™, LOCTITE®) were applied using a high-resolution dispenser.

There were four types of electrode materials: (1) ~100-nm thick Au film with 5-nm thick Ti seed layer deposited on flexibles (e.g., PET sheets, Hi-Fi Industrial Film Ltd.) via an electron beam evaporator (Kurt J. Lesker); (2) ~100-nm thick Pt film sputtered onto flexibles using a sputtering coater (Emitech K575X, Quorum Technologies Ltd.) with a 99.99% purity Pt target (Mi-Net Technology Ltd.); (3 and 4) Porous carbon meshes (A: Sigracet® 39 AA, SGL Carbon; B: GF020 graphite felt, The Fuel Cell Store).

### Device characterizations

The metal content in the sample was determined by Thermo Fisher Scientific iCAP7400 Duo ICP-OES spectrometer with *Qtegra* software, calibrated against standard curves constructed from a series of dilutions of commercial ICP standards with a 2% nitric acid solution.

Absorbance spectra were acquired using a UV–visible–NIR spectrometer (Avantes BV).

The sheet resistance of electrode materials was measured using a four-point probe, custom-built in-house with Keysight, and calibrated using an Ag foil (265535, Sigma-Aldrich®) with a resulting resistance of ~1.3 Ω/sq.

Images of SEM (scanning electron microscope), AFM (atomic force microscope), and optical microscope were captured using a Leo 1530 VP system (Gemini®), AFM (Bruker Bioscope Resolve Atomic Force Microscope with ScanAsyst-Air probe silicon tips) and Elipse LV 100ND (Nikon Metrology NV), respectively.

Fluid pressure, flow rate, and pH were recorded using a pressure sensor (PRESS-S-000, PendoTECH), a liquid flow sensor (SLF3S-0600F, Sensirion AG), and a pH sensor (METTLER TOLEDO), respectively.

The membrane activation was characterized under 1 V and 0.1 mL/min of 0.2 M sodium ferrocyanide solution. The control device was assembled layer-by-layer by carefully applying liquid adhesives on the external surfaces of the microfluidics, ensuring that there were no residues on the membrane.

The quantity of drug delivery was analyzed using a Spark® multimode microplate reader.

A PalmSen® 4 potentiostat was used as a power supply, and to perform measurements of impedance and current response.

The methylene blue delivery into gel brain phantoms was imaged using the setup as reported in ref. ^47^.

Accelerated aging tests were conducted using an LSE Benchtop Shaking Incubator (Corning®) by increasing the ambient temperature^55,56^, where the Arrhenius equation was employed for the calculation of the acceleration rate^57^. The pH test over days was conducted at 45 °C using an accelerated aging setup.

## Supplementary information

Supplemental Material File #1
