# Supplementary material for: Toolkit for integrating millimeter-sized microfluidic biomedical devices with multiple membranes and electrodes
Source: Microsyst Nanoeng. 2025 Feb 27;11:33. doi: 10.1038/s41378-025-00871-0 (PMC11865549; doi:10.1038/s41378-025-00871-0)
Supplement: Supplementary file 1 — Supplemental Material File #1 [file 41378_2025_871_MOESM1_ESM.docx]

**Toolkit for Integrating Millimeter-Sized Microfluidic Biomedical Devices with Multiple Membranes and Electrodes**

Xudong Tao^a†^, Tobias E. Naegele^a†^, Etienne Rognin^b^, Niamh Willis-Fox^b^, Poppy Oldroyd^a^, Chaoqun Dong^a^, Stefany Kissovsky^a^, Antonio Dominguez-Alfaro^a^, Santiago Velasco-Bosom^a^, Ronan Daly^b^, George G. Malliaras^a^*

a: Electrical Engineering Division, Department of Engineering, University of Cambridge, Cambridge, CB3 0FA, UK

b: Institute for Manufacturing, Department of Engineering, University of Cambridge, CB3 0FS, UK

†: Contributed equally.

*: Corresponding author, George Malliaras [gm603@cam.ac.uk](mailto:gm603@cam.ac.uk)

Supplementary Information


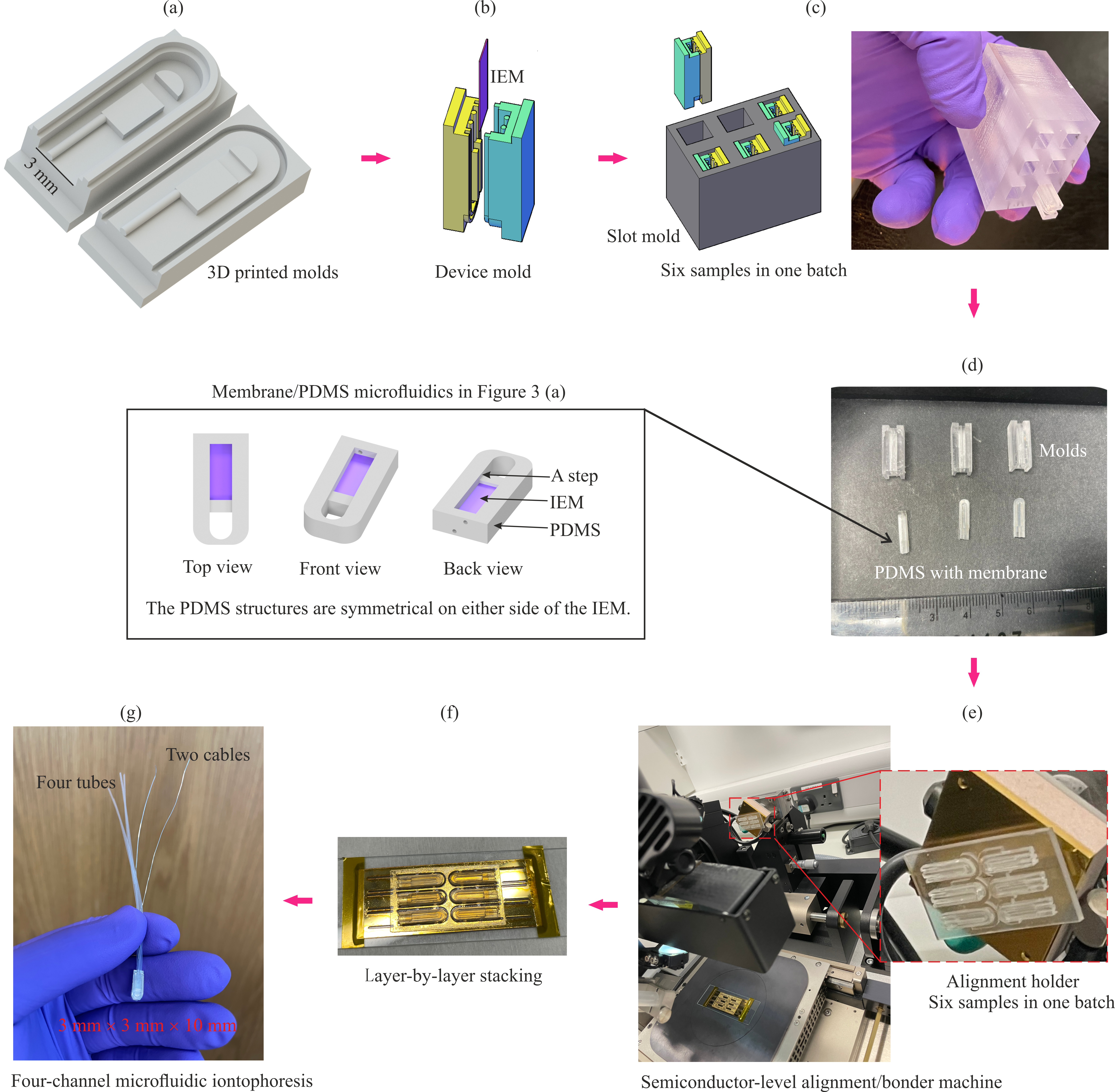


Figure S1. Fabrication process of a redox flow iontophoretic device with cross-section U-shape design of four-channel microfluidics (dimension: 3 mm × 3 mm × 10 mm): (a) 3D printing molds, (b) membrane into device molds, (c) device molds into a slot mold, (d & e) batch-to-batch processing using an aligner, (f) layer-by-layer stacking for integration, (g) tube/cable connection. All steps can be processed batch-to-batch, enabling scalable production.

**Figure S2**. (a) Deformation of the IEM membrane in a dry state; (b) Porous membrane; (c) Images of PDMS residuals with varying RIE etching time (4, 43 and 180 minutes); (d) Setup for characterization of membrane efficiency for an in-plane U-shape device; (e) Setup for characterization of burst pressure.


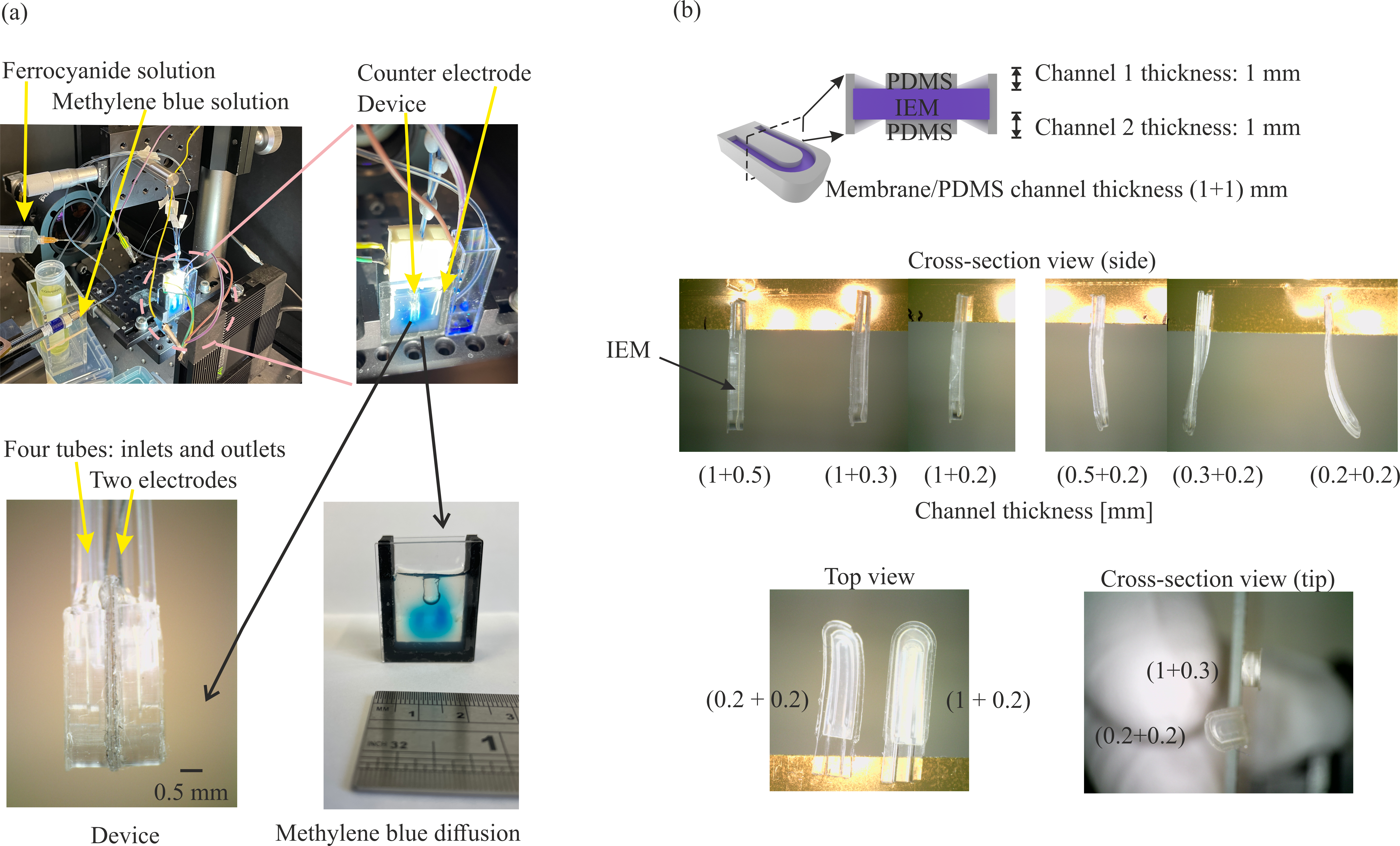


**Figure S3**. (a) Characterization setup for delivery efficiency using methylene blue: the device and the diffusion of methylene blue; (b) Effect of channel thickness on IEM deformation.


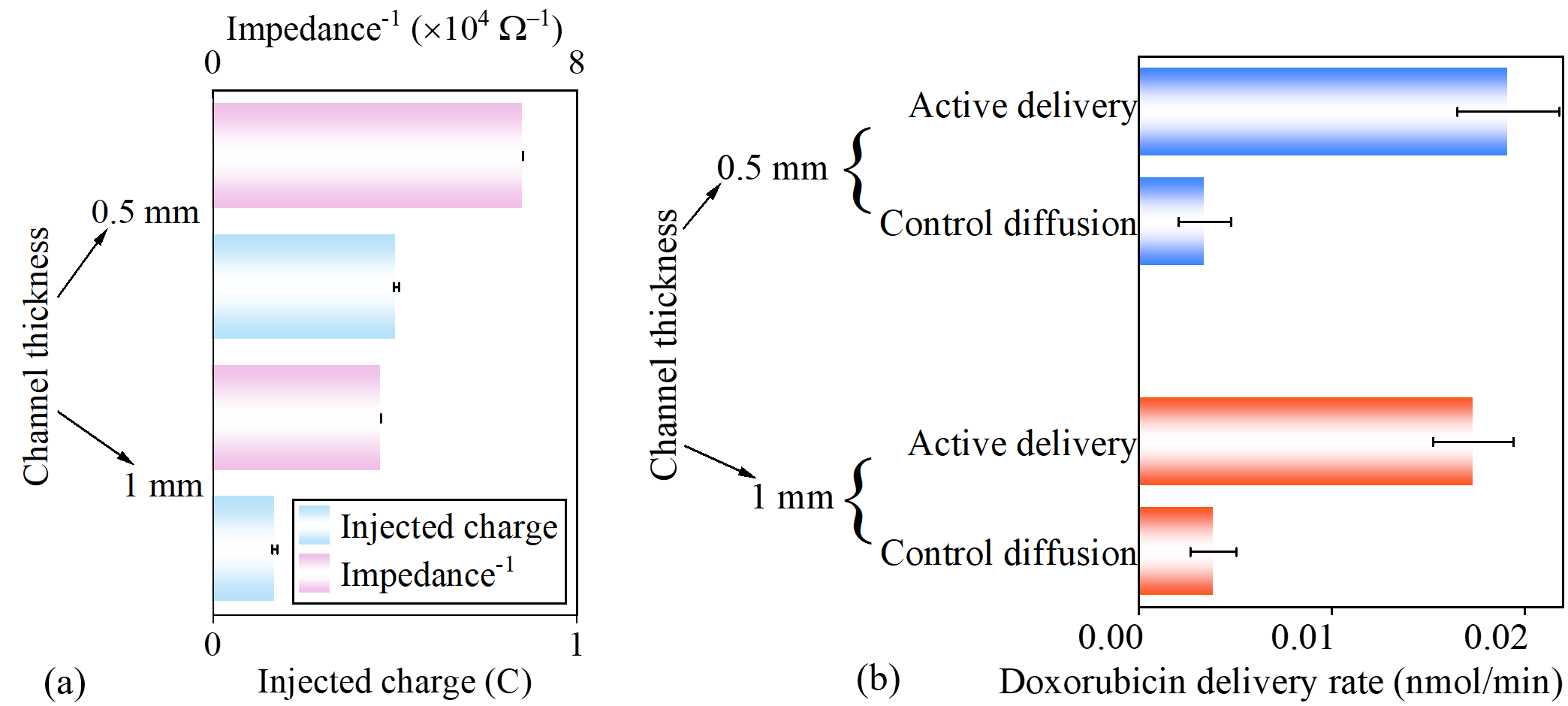


**Figure S4**. Influence of channel thickness (1 mm and 0.5 mm) on (a) injected charge/ impedance, and (b) drug delivery rate.


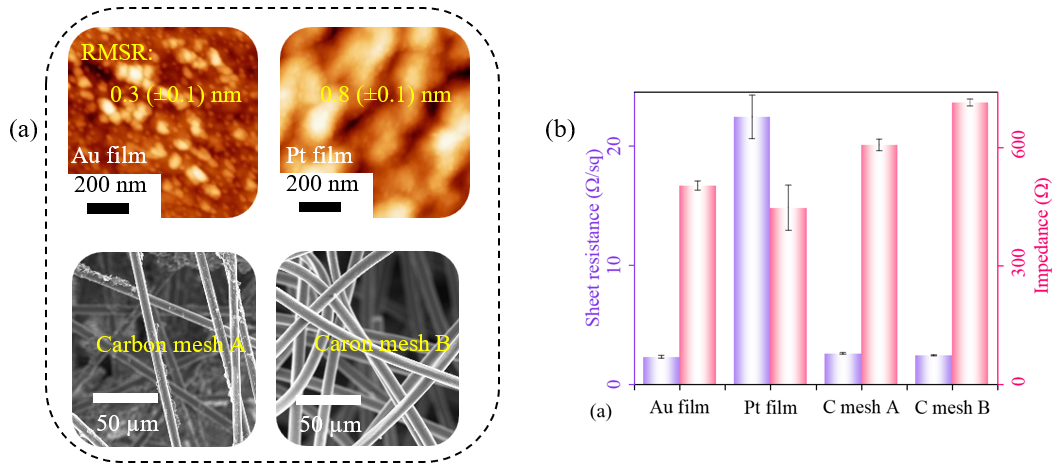


**Figure S5**. (a) Surface morphologies of thin films (Au and Pt) and bulk materials (carbon mesh); (b) Sheet resistance and impedance in 100 mM NaCl electrolyte of four different materials.


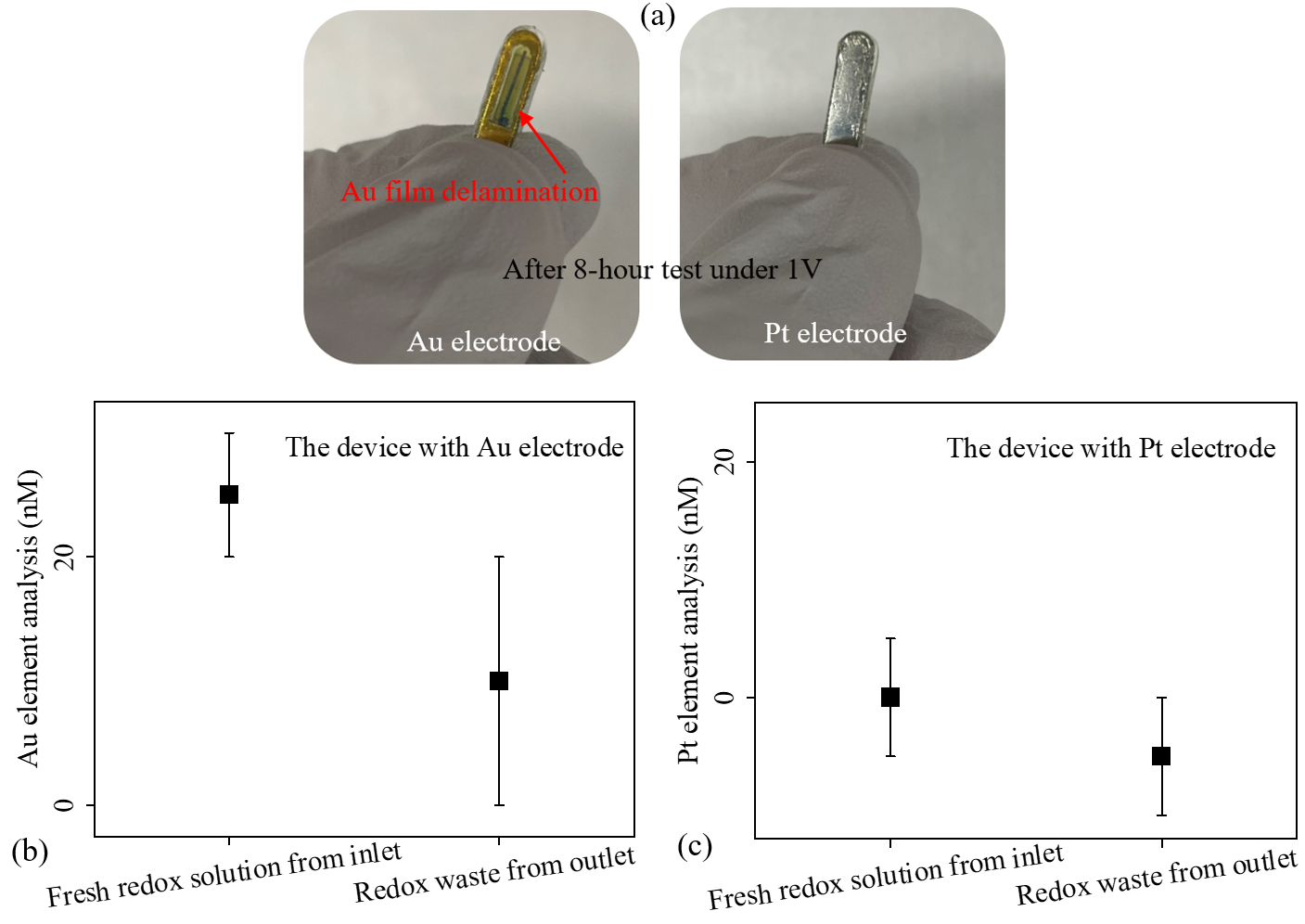


**Figure S6**. (a) The redox iontophoresis with Au and Pt electrodes (after an 8-hour test under 1V, the Au electrode was consumed/delaminated while the Pt electrode did not have this delamination issue); (b & c) Elemental analysis of the redox waste from the device outlet.


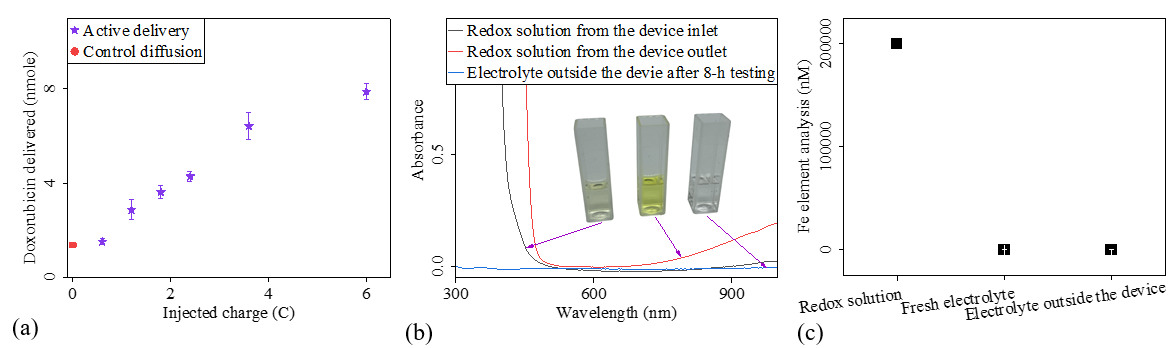


**Figure S7**. (a) Plot of injected charges vs. drug delivery. Error bars represent measurements from three identical devices; (b) UV-Vis analysis of the fresh redox electrolyte, the redox waste, and the NaCl electrolyte outside the device after 8-hour testing; (c) Elemental analysis using ICP-OES to check the diffusion of ferrocyanide outside of the device. Error bars represent measurements from three identical samples.


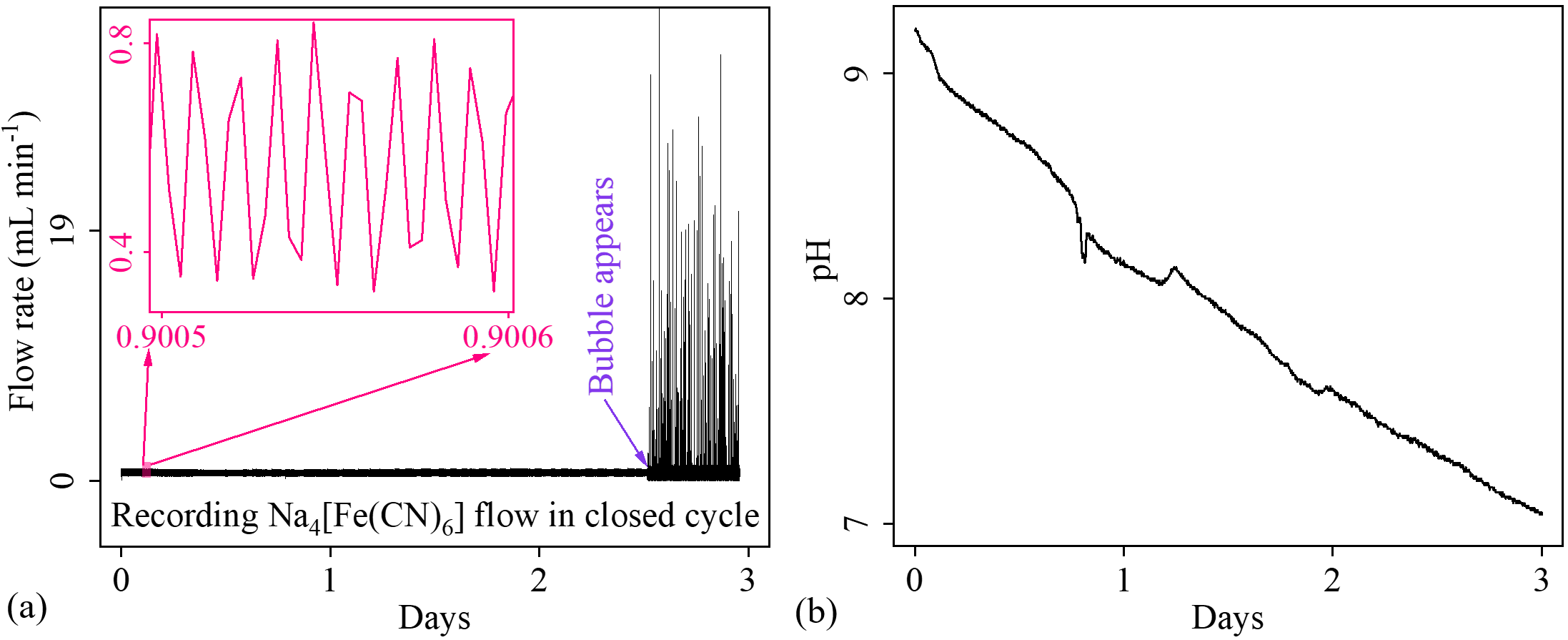


**Figure S8**. (a) Flow rate of ferrocyanide fluid over the 3-day experiment; (b) pH recording of the redox solution over time.

**Bubble assumption**

The pH drop could result from the movement of OH^-^ through the anion IEM to the drug channel to balance the pH between the redox solution (pH = 9) and the drug solution (pH = 3). Additionally, the OH^-^ in the redox channel could compete with the Cl^-^ to go through the anion IEM at the reduction side, thereby affecting the pH. Although it is unlikely to occur, the equilibrium state of the Donnan condition [1, 2] might be disrupted over days due to a significant change in the electrolyte concentration near the IEM, thus enabling the transport of co-ions (i.e. H^+^) across the anion IEM. In relation to bubble formation, it is improbable that hydrogen and oxygen are generated through hydrolysis, given that the applied voltage is significantly below 1V. The formation of HCN gas is also unlikely because it requires a pH of 1 - 2 [3], which is far from the pH in our case. As pH drops and Cl^-^ continues to increase in the redox channel at the positively charged side, the Cl_2_ gas could be generated. Therefore, the pH of the redox solution should be carefully controlled e.g. by increasing the pH of the drug (Ferrocyanide/ferricyanide is stable under a high alkaline condition [4]). In practical terms, both redox and drug solutions should be refilled periodically, similar to the intrathecal drug delivery system for chronic pain (e.g. Hospital at Home for intrathecal pump refills, [5]).


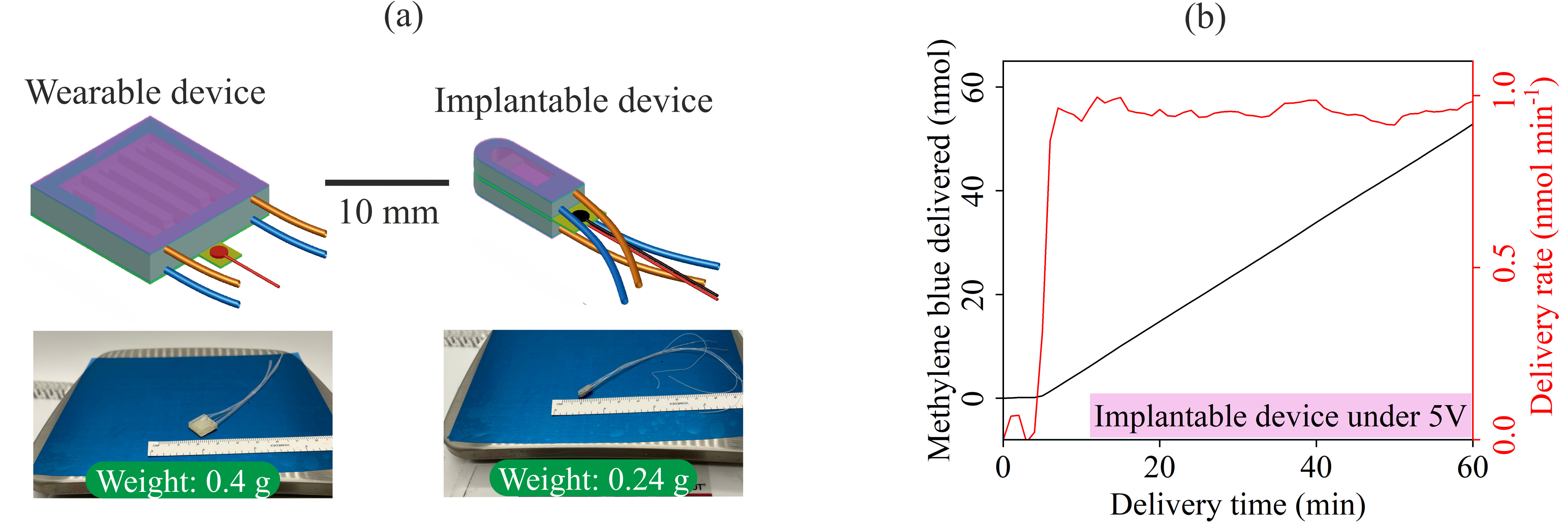


**Figure S9**. (a) Various geometries of redox flow iontophoresis devices; (b) Single-pass delivery of methylene blue under 5V applied.


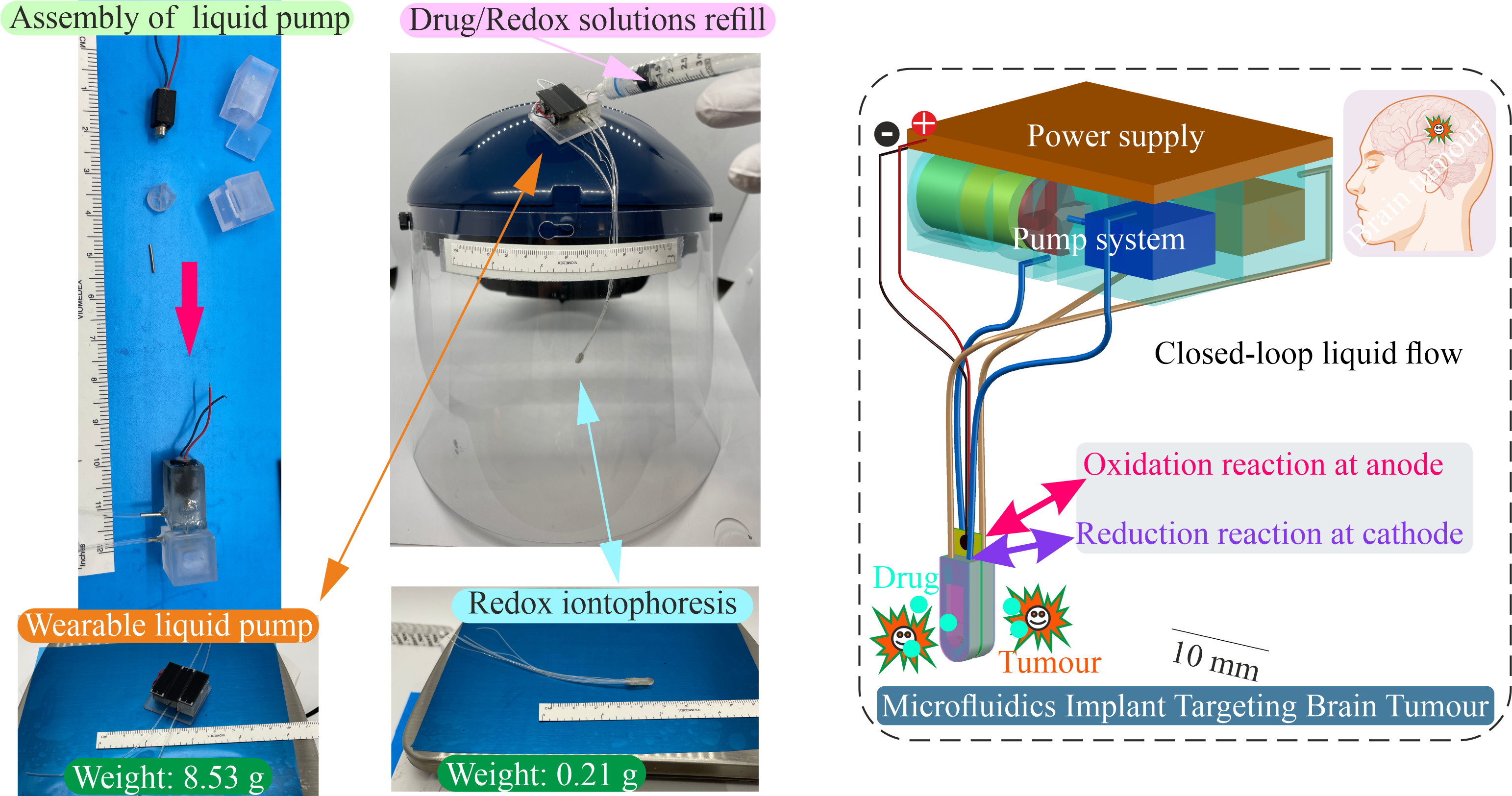


**Figure S10**. Integration of wearable pump with redox flow iontophoresis for Hospital at Home treatment.


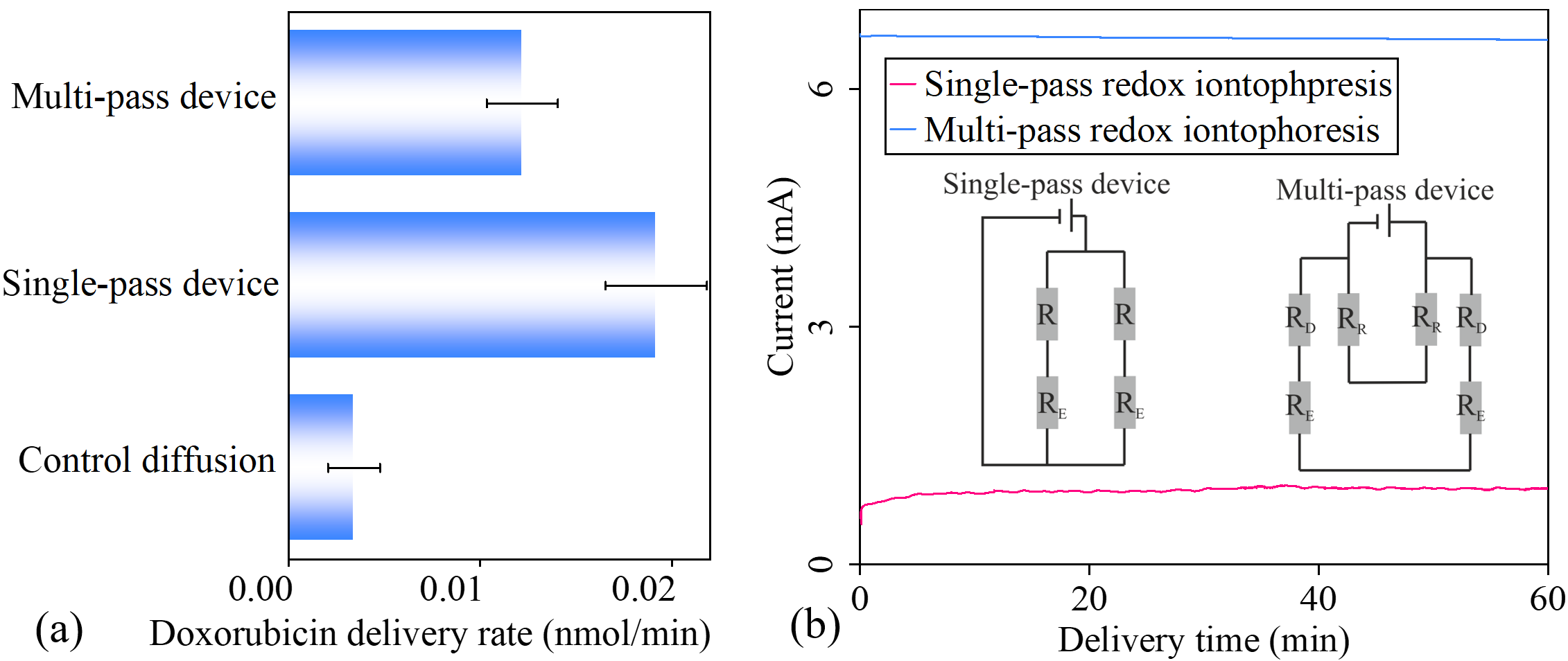


**Figure S11**. (a) Comparison of drug delivery between single-pass and multi-pass devices; (b) Current response and electrical circuit diagrams for single-pass and multi-pass devices. R denotes the resistance of one half of the device, comprising one redox channel (R_R_) and one drug channel (R_D_), while R_E_ denotes the resistance of the electrolyte. It is noted that there is also skin resistance for the single-pass case.


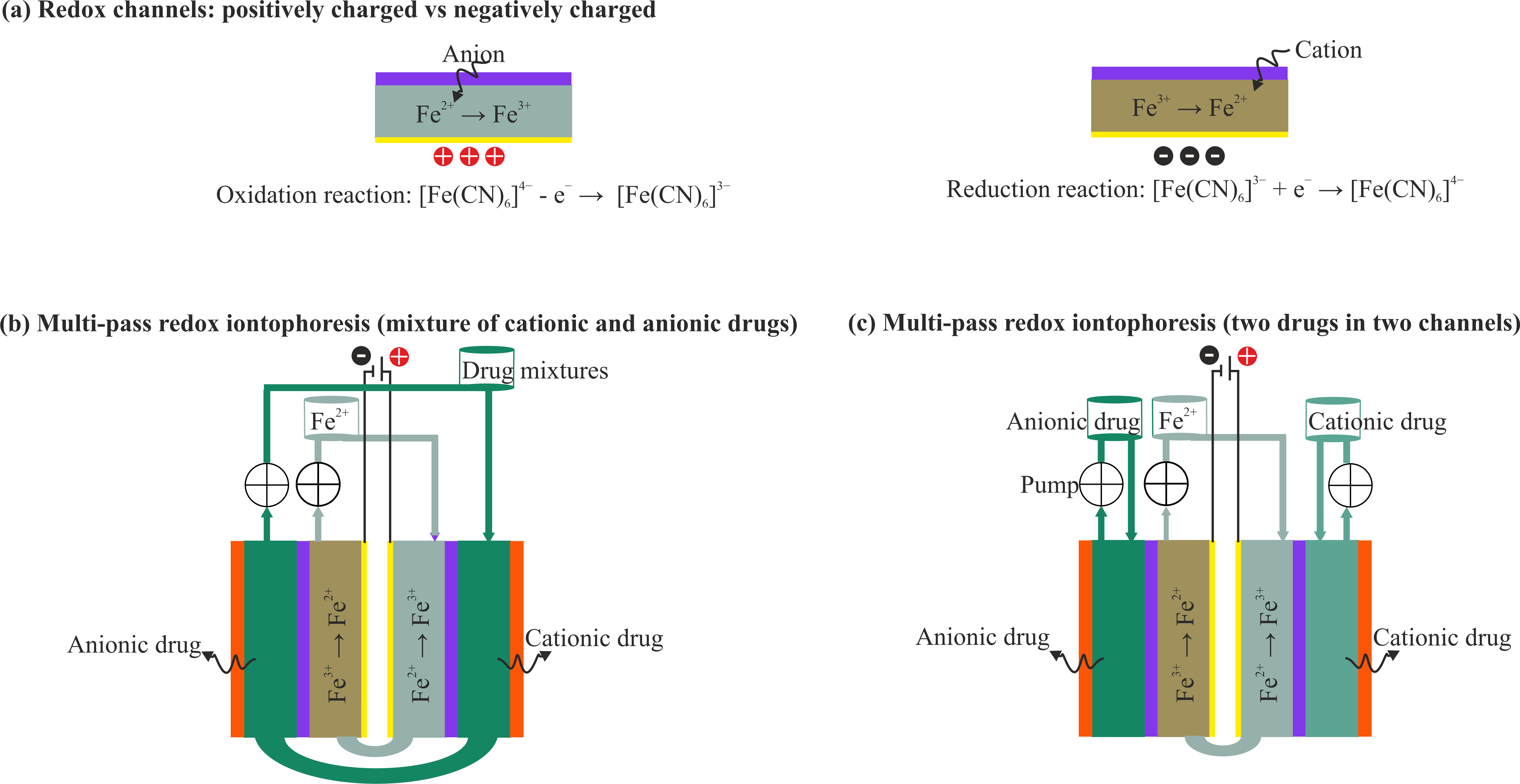


**Figure S12**. (a) Redox reactions occurring at the negatively charged side and positively charged side; (b & c) Multi-pass redox iontophoresis for two-type drug delivery, either by mixing them (b) or by separating them into two channels (c), depending on whether the drugs interact with each other.

**Reference**

[1] Luo, T.; Abdu, S.; Wessling, M. , Selectivity of ion exchange membranes: A review, *Journal of Membrane Science.* 2018*, 555*, 429-454, <https://doi.org/10.1016/j.memsci.2018.03.051.>

[2] Aydogan Gokturk, P.; Sujanani, R.; Qian, J.; Wang, Y.; Katz, L. E.; Freeman, B. D.; Crumlin, E. J. , The Donnan potential revealed, *Nature Communications.* 2022*, 13*, 5880, <https://doi.org/10.1038/s41467-022-33592-3.>

[3] Domingo, P. L.; García, B.; Leal, J. M. , Acid-base behaviour of the ferricyanide ion in perchloric acid media. Spectrophotometric and kinetic study, *Canadian Journal of Chemistry.* 1990*, 68*, 228-235, <https://doi.org/10.1139/v90-030.>

[4] Fell, E. M.; De Porcellinis, D.; Jing, Y.; Gutierrez-Venegas, V.; Gordon, R. G.; Granados-Focil, S.; Aziz, M. , Long-Term Stability of Ferri/Ferrocyanide As an Electroactive Component for Redox Flow Battery Applications: On the Origin of Apparent Capacity Fade, *Meeting Abstracts (Electrochemical Society).* 2022*, MA2022-02*, 1726, <https://iopscience.iop.org/article/10.1149/MA2022-02461726mtgabs.>

[5] Goudman, L.; De Smedt, A.; Huygens, R.; Noppen, M.; Vanschoenwinkel, M.; Hatem, S. M.; Moens, M. , Hospital at Home for Intrathecal Pump Refills: A Prospective Effectiveness, Safety and Feasibility Study, *Journal of Clinical Medicine.* 2021*, 10*, 5353, <https://doi.org/10.3390/jcm10225353.>
